# Dominant-Negative Form of SIGIRR: SIGIRR^ΔE8^ Promotes Tumor Growth Through Regulation of Metabolic Pathways

**DOI:** 10.1089/jir.2022.0095

**Published:** 2022-09-15

**Authors:** Malgorzata Bodaszewska-Lubas, Yun Liao, Aneta Zegar, Oskar Szelest, Jurek Dobrucki, Katarzyna Bulek

**Affiliations:** ^1^ Department of Immunology and Faculty of Biochemistry, Biophysics, and Biotechnology, Jagiellonian University, Krakow, Poland.; ^2^ Department of Inflammation and Immunity, Cleveland Clinic, Lerner Research Institute, Cleveland, Ohio, USA.; ^3^ Department of Cell Biophysics, Faculty of Biochemistry, Biophysics, Biotechnology, Jagiellonian University, Krakow, Poland.

**Keywords:** SIGIRR, tumor, xenograft

## Abstract

Colorectal carcinoma is the leading cause of cancer-related death. Previously we have shown that tumor suppressor single immunoglobulin interleukin-1-related receptor (SIGIRR) is frequently inactivated in human colorectal cancer by the increased expression of a novel SIGIRR isoform (SIGIRR^ΔE8^). SIGIRR^ΔE8^ showed increased retention in the cytoplasm and loss of complex glycan modification compared to the full-length SIGIRR. Now we found that the arginine residues located in the C-terminus of SIGIRR^ΔE8^ serve as an endoplasmic reticulum retention signal and are required for resident protein ribophorin 1 (RPN1) interaction. In addition, we found that SIGIRR^ΔE8^ exerts a direct impact on cell metabolism through interaction with the adenosine triphosphate synthase in the colorectal cancer cells. SIGIRR^ΔE8^ expression promoted the metabolic shift through upregulation of mammalian target of rapamycin signaling pathway and dysregulation of mitochondrial function to promote survival and proliferation of colon cancer cells in xenograft model.

## Introduction

Colorectal carcinoma (CRC) is the leading cause of cancer-related death in the world (Ferlay et al, 2015). The development of CRC follows a stepwise normal-adenoma-cancer progression (Fearon and Vogelstein, 1990). The transitions from normal mucosa to adenomatous polyp to cancer are marked by both genetic and epigenetic alterations, including DNA methylation (Markowitz and Bertagnolli, 2009). Inactivation of tumor suppressor genes by epigenetic alterations such as DNA methylation is a fundamental contributor to tumor development (Grady, 2005; Grady and Markowitz, 2002; Jaenisch and Bird, 2003; Samowitz, 2008; Samowitz et al, 2007). Although epigenetic disruption was originally thought to only affect mRNA transcription, emerging evidence shows that it also regulates alternative splicing (Allo et al, 2010; Luco et al, 2011; Zhou et al, 2012). A critical step in the pathogenesis of CRC is the inactivation of tumor suppressor genes.

SIGIRR [single immunoglobulin interleukin (IL)-1-related receptor, also named as TIR8] represents a unique subgroup of the toll-like receptor (TLR)/IL-1R superfamily, with a single Ig extracellular domain and a TIR intracellular domain (Garlanda et al, 2004; Thomassen et al, 1999; Wald et al, 2003). We and others have shown that SIGIRR functions as a negative regulator of IL-1, IL-33, lipopolysaccharide, and CpG signaling through its interaction with the IL-1R, ST2 (receptor for IL-33), TLR4, and TLR9 complex (Bulek et al, 2009; Drexler et al, 2010; Garlanda et al, 2004, 2007; Gong et al, 2010; Gulen et al, 2010; Lech et al, 2007; O'Neill, 2003; Wald et al, 2003; Xiao et al, 2007; Zhang et al, 2011). SIGIRR inhibits the IL-1R subfamily and TLR signaling pathways through different mechanisms (Qin et al, 2005). The extracellular Ig domain interferes with the heterodimerization of the immunoglobulin domains of the receptor subunits of the IL-1R subfamily, whereas the intracellular TIR domain inhibits both the IL-1R subfamily and TLR signaling by attenuating the recruitment of receptor proximal signaling components to the receptor.

We have previously shown that SIGIRR functions as a tumor suppressor in both murine models of colitis-associated tumor and in spontaneous CRC model in *Apc*^min^ mice (Xiao et al, 2007, 2010; Zhao et al, 2011). SIGIRR is highly expressed in colon epithelial cells and negatively regulates TLR/IL-1R signaling (Garlanda et al, 2007; Qin et al, 2005; Thomassen et al, 1999; Wald et al, 2003), restricting the responsiveness of the gut epithelium to TLR ligands (from the microflora).

Recently, we have demonstrated that SIGIRR is frequently inactivated in human colorectal cancer by the increased expression of a novel SIGIRR isoform (SIGIRR^ΔE8^) (Zhao et al, 2015). SIGIRR^ΔE8^ transcript is generated by an alternative splicing event that excludes the eighth exon of the SIGIRR gene. SIGIRR^ΔE8^ functions as a dominant negative mutant that traps the full-length SIGIRR protein in the endoplasmic reticulum (ER) through interaction with the ER resident protein ribophorin 1 (RPN1), preventing its modification by complex glycan and membrane localization. Integrated analysis of exon and RNA sequencing data from 68 pairs of normal and colon cancer samples indicates that SIGIRR^ΔE8^ alternative splicing is independent of detected genetic mutations, suggesting an epigenetic mechanism underlying the splicing (Zhao et al, 2015). The aim of the current study was to investigate the ER retention and the mechanism of SIGIRR^ΔE8^-mediated tumor promotion.

## Materials and Methods

### Animals

All animal experiments were conducted in accordance with Institutional Animal Care and Use Committee (IACUC) guidelines at the Cleveland Clinic Lerner Research Institute. Adult (8-week old to 16-week old) mice were used. All mice were maintained in a specific pathogen-free barrier facility under a strict 12-h light/12-h dark cycle and were fed the same standard autoclaved chow diet. NOD/scid gamma (NSG) mice were supplied by the Biological Resource Unit at the Cleveland Clinic from a colony maintained in-house.

### Generation of inducible cell lines

The colon cancer cell line HT-29 was used to generate the clones in this study. To generate the SIGIRR^ΔE8^-inducible clone, V5-tagged human SIGIRR^ΔE8^ cDNA under the control of Tet-responsive element was transduced into the HT-29 cells along with tetracycline transactivator cDNA using lentiviral infection. The infected cells were dispersed into single cells and derived into independent clones. The clones are validated for doxycycline-dependent expression of V5-tagged SIGIRR^ΔE8^ with Western blot, and 5 independently validated clones were pooled for subsequent experiments.

### Xenograft tumor model

To investigate tumor growth difference, each flank of NSG mouse was subcutaneously implanted with 0.5 million engineered HT-29 cells in 100 μL cold phosphate-buffered saline (PBS)−Matrigel (Corning) mixture (1:1 ratio). Implanted mice were randomly assigned to each treatment group. SIGIRR^ΔE8^ expression was continuously induced by Doxycycline in drinking water (2 mg/mL with 1% sucrose). The control mice were given pure water. Tumor size was measured using caliber and was initiated 3 days after implantation. Tumor volume was calculated as length/2 × (width)^2^.

### Plasmids

DNA encoding N-terminal FLAG-tagged SIGIRR was cloned into the vector pCDNA3.1 (+) purchased from Invitrogen. Site-directed mutagenesis was performed on N-terminal FLAG-tagged single immunoglobulin and toll-interleukin 1 receptor (SIGIRR) on pcDNA3.1 (+), using a QuikChange Kit (Agilent Technologies, Wilmington, DE) according to the manufacturer's instruction. In all cases, the asparagine was mutated to serine on the plasmid.

### Luciferase reporter assays

HeLa cells were transiently transfected using Lipofectamine3000 with nuclear factor kappa B (NFκB) luciferase reporter plasmid following the manufacturer's protocol. Empty vector was used to ensure all wells received equal amounts of DNA. Twenty-four hours after transfection, cells were stimulated with 1 ng/mL IL-1β for 8 h. Cells were lysed, and luciferase activity was assessed using Reporter lysis buffer and Luciferase Assay Reagent (Promega). All results reported are technical triplicates representing at least 3 independent experiments.

### *In situ* biotinylation, immunoprecipitation

Biotinylation was performed by rinsing transfected cells with cold PBS followed by incubation with freshly prepared 10 mM sulfo-NHS-biotin dissolved in cold PBS for 2 h. The labeling process was stopped by siphoning away the labeling reagent and quenching with 100 mM glycine dissolved in PBS. The cells were then harvested and lysed for lysates. The supernatant was collected for Western blotting. Coimmunoprecipitation was performed by incubating cell lysates with antibodies and protein A beads or avidin conjugated beads at 4°C overnight. Precipitated protein-beads complex was washed with lysis buffer followed by elution with 2 × sodium dodecyl sulfate–polyacrylamide gel electrophoresis loading buffer.

### Transfection

Transfection was performed using Lipofectamine3000 according to the manufacturer's protocol.

### Imaging

The transfected cells and controls were fixed with 4% paraformaldehyde (PFA) and stained with anti-hemagglutinin tag primary antibodies followed by corresponding secondary antibodies and Hoechst 33342. Cells fixed on the glass coverslips were imaged using Leica SP5 SMD confocal microscope, equipped with 60 × HCX PL APO CS NA 1.4 lens. Excitation was 488 nm (for Alexa 488) and 405 nm (for Hoechst). Emission was recorded in 415–460 nm and 500–535 nm ranges. Images shown in Fig. 2B were histogram stretched.

### Western blot and immunoprecipitation

Cells were lysed on ice using lysis buffer (0.5% Triton X-100, 50 mM Tris-HCl (pH 7.4), 150 mM NaCl, 12.5 mM β-glycerophosphate, 1.5 mM MgCl2, 10 mM NaF, 2 mM dithiothreitol (DTT), 2 mM sodium orthovanadate, 2 mM ethylene glycol tetraacetic acid, and Protease Inhibitor Cocktail (Roche)) followed by centrifugation at 12,000*g* for 20 min at 4°C. Supernatants were carefully collected for Western blot analysis or coimmunoprecipitation. For immunoprecipitation, cell lysates or supernatants were incubated overnight at 4°C with targeting antibody and protein A/G Sepharose beads (GE Healthcare Life Sciences), followed by extensive washes with lysis buffer. Precipitates were eluted by SDS loading buffer and analyzed by the Western blot. For immunoprecipitation analysis TrueBlot secondary antibodies were used (Rockland).

### Antibodies

The following antibodies are used in Western blot:

**Table:** 

Protein	Clone no.	Catalog no.	Company	Usage
β-Actin	8H10D10	3700	Cell Signaling	1:1,000 for WB
α-Tubulin	N/A	2144	Cell Signaling	1:1,000 for WB
GAPDH	D4CR6	97166	Cell Signaling	1:1,000 for WB
Flag	M2	F3165	Sigma	1:1,000 for WB/1:100 for immunoprecipitation
DYKDDDDK	D6W5B	14793	Cell Signaling	1:1,000 for WB/1:100 for immunoprecipitation
Myc-tag	9B11	2276	Cell Signaling	1:1,000 for WB/1:1,000 for immunoprecipitation
Myc-tag	71D10	2278	Cell Signaling	Cell Signaling 1:1,000 for WB/1:1,000 for immunoprecipitation
V5	E10/V4RR	MA5-15253	Invitrogen	1:1,000 for WB/1:100 for immunoprecipitation
V5	D3H8Q	13202	Cell Signaling	1:1,000 for WB/1:100 for immunoprecipitation
SIGIRR	N/A	AF990	R&D	1:1,000 for WB/1:100 for immunoprecipitation
SIGIRR	EPR12638	ab177937	Abcam	1:1,000 for WB
p-IKKα/β	16A6	2697	Cell Signaling	1:1,000 for WB
*P*-p65	93H1	3033	Cell Signaling	1:1,000 for WB
*P*-IκBα	14D4	2859	Cell Signaling	1:1,000 for WB
p-JNK1/2	G9	9255	Cell Signaling	1:1,000 for WB
p-ERK1/2	D13.14.4E	4370	Cell Signaling	1:1,000 for WB
*P*-p38	D3F9	4511	Cell Signaling	1:1,000 for WB
p-mTOR	D9C2	5536	Cell Signaling	1:1,000 for WB
p-90RSK	D3H11	11989	Cell Signaling	1:1,000 for WB
*P*-p70S6K	N/A	9204	Cell Signaling	1:1,000 for WB
*P*-S6	D57.2.2E	4858	Cell Signaling	1:1,000 for WB
p-4E-BP1	236B4	2855	Cell Signaling	1:1,000 for WB
APT5A1	N/A	18023	Cell Signaling	1:1,000 for WB
Na+K+ATPASE	N/A	3010	Cell Signaling	1:1,000 for WB
Rabbit TrueBlot	eB182	18-8816-31	Rockland	1:1,000 for WB
Mouse TrueBlot	eB144	18-8817-30	Rockland	1:1,000 for WB
Goat TrueBlot	eB270	18-8814-31	Rockland	1:1,000 for WB

### Quantitative real-time polymerase chain reaction

In all experiments, RNA was extracted with TRIzol (Invitrogen) followed by reverse transcription with SuperScript II reverse transcriptase (Life Technologies, Carlsbad, CA) according to the manufacturer's instruction. Real-time polymerase chain reaction analysis was performed using SYBR Green Master mixture (Agilent Technologies). Primer sequences were MMP8 forward-5′ CCAGCACCTATTCACTACCTC 3′ and reverse-5′ AGCATCAAATCTCAGGTGGG3′; TNFα forward-5′ TCAGCAAGGACAGCAGAG and reverse-5′ GTATGTGAGAGGAAGAGAACC; DEFB4A forward-5′ ATCAGCCATGAGGGTCTTGT and reverse-5′ GAGACCACAGGTGCCAATTT; CYCLIN D1 forward-5′ CCGTCCATGCGGAAGATC and reverse-5′ GAAGACCTCCTCCTCGCACT; MCP-1 (CCL2) forward-5′ CCCCAGTCACCTGCTGTTAT and reverse-5′ -TGGAATCCTGAACCCACTTC; GAPDH forward-5′ GCAAATTCCATGGCACCGT and reverse-5′ TCGCCCCACTTGATTTTGG; β-ACTIN forward-5′ GTCGGTATGGGTCAGAAAG and reverse-5′ CTCGTTGTAGAAGGTGTGG.

### Immunohistochemistry and immunofluorescence

Mouse tissue was fixed with 10% formalin overnight and kept in 70% ethanol at 4°C until processed into paraffin tissue blocks by the Imaging Core at Lerner research institute of Cleveland Clinic. Paraffin sections were subjected to heat-induced epitope retrieval, as recommended by the antibody manufacturer before staining. Deparaffinized, epitope-retrieved sections were blocked with 10% normal goat serum (Life Technologies; no. 50062Z) and then incubated with primary antibody overnight. Next day, the sections were washed with 0.05% Tween PBS followed by the incubation with secondary antibody and streptavidin-horseradish peroxidase (HRP) for immunohistochemistry or fluorescence-conjugated secondary antibody for immunofluorescence. Staining was visualized with HRP-substrate chromogen DAB (BD Pharmingen) or with confocal microscopy. The following primary antibodies were used for staining presented in the study: Ki67 (Cell Signaling Technology 12202, 1:200).

### BrdU labeling

SIGIRR^ΔE8^-inducible HT-29 (untreated or doxycycline treated to induce SIGIRR^ΔE8^ expression) was labeled with 10 μM of BrdU (Bromodeoxyuridine/5-bromo-2′-deoxyuridine) for 6 h followed by fixation with 4% PFA, DNA hydrolysis with 2N HCl for 30 min at room temperature. HCl was removed and neutralized with 0.1 M sodium borate buffer pH 8.5 for 30 min at room temperature followed by an immunofluorescence staining with anti-BrdU antibody (R&D; cat no. MAB7225, clone no. BU-1, 25 μg/mL).

### Metabolic profiling

Extracellular flux (XFp) assays (Seahorse XF Glycolysis Stress Test Kit and Seahorse XF Cell Mito Stress Test Kit; Seahorse Bioscience, Chicopee, MA) were used to evaluate the oxygen consumption rate (OCR) and extracellular acidification rate (ECAR) according to the manufacturer's instructions. Briefly, the cells were plated in Dulbecco's modified Eagle's medium into Seahorse XF Cell Culture Microplates (1.2 × 10^4^ cells/well) and incubated overnight. Before assays, cells were washed with Seahorse Medium supplemented with glucose, glutamine, and pyruvate (pH 7.4) for OCR and pyruvate (pH 7.4) for ECAR. The OCR and ECAR were assessed after the addition of oligomycin, carbonyl cyanide 4-(trifluoromethoxy)phenylhydrazone, rotenone/antimycin A and glucose, oligomycin, 2-deoxyglucose (2-DG), respectively, with the Seahorse XFp Analyzer. The assay results obtained were normalized to the total number of cells in a given well.

### Liquid chromatography–mass spectrometry/mass spectrometry method

Immunoprecipitation (IP) and control IgG samples were fractionated on an SDS-Page gel and entire lane cut into 2 bands, which were subjected to in-gel digestion. Gel bands were divided into small pieces, washed with water, and dehydrated in acetonitrile. Bands were reduced with DTT and alkylated with iodoacetamide before in-gel digestion, achieved by adding 5 μL of 10 ng/μL trypsin in 50 mM ammonium bicarbonate, and incubating overnight at room temperature. Formed peptides were extracted from polyacrylamide into 2 aliquots of 30 μL 50% acetonitrile with 5% formic acid. Extracts were combined and evaporated to <10 μL by Speedvac and resuspended in 1% acetic acid to a final volume of ∼30 μL for liquid chromatography–mass spectrometry analysis.

Digested peptides were analyzed on a Thermo Fisher Scientific UltiMate 3000 high performance liquid chromatography (HPLC) system (Thermo Fisher Scientific, Bremen, Germany) interfaced with either a ThermoFisher Orbitrap Elite or ThermoFisher Scientific Orbitrap Fusion Lumos Tribrid mass spectrometer (Thermo Scientific). Liquid chromatography was performed before MS/MS analysis for peptide separation. The HPLC column used was a Dionex 15 cm × 75 μm Acclaim PepMap C18, 2 μm, 100Å reversed-phase capillary chromatography column. Five microliter volume of peptide extracts were injected and eluted from the column by a 90 min acetonitrile/0.1% formic acid gradient at a flow rate of 0.30 μL/min and introduced to the mass spectrometer source on-line. Digests were analyzed using data-dependent multitask capability of the instrument acquiring full scan mass spectra on a Fourier Transform orbitrap analyzer to determine peptide MWs and collision-induced dissociation MS/MS product ion spectra with an ion-trap analyzer to determine the amino acids (aa) sequence in successive instrument scans.

To do label-free quantitative and qualitative proteomic analysis, data were searched using X! Tandem (The GPM) and Sequest (bundled into Proteome Discoverer 2.2; Thermo Fisher Scientific, San Jose, CA). The database used in these searches corresponds to the mouse SwissProtKB database. The parameters used for these searches include enzyme specific-trypsin with a maximum of 2 missed cleavages, carbamidomethyl (C) as a fixed modification, oxidation of methionine and protein N-terminal acetylation as variable modifications, peptide mass tolerance of 10 ppm, and fragment ion mass tolerance of 0.6 Da. Scaffold (version Scaffold 4.8.9; Proteome Software, Inc., Portland, OR) was used to validate MS/MS-based peptide and protein identifications.

Peptide identifications were accepted if they could be established at >0.0% probability by the Peptide Prophet algorithm (Keller et al, 2002) with Scaffold delta-mass correction. Protein identifications were accepted if they could be established at >99.9% probability and contained at least 2 identified peptides. Protein probabilities were assigned by the Protein Prophet algorithm (Nesvizhskii et al, 2003). Proteins that contained similar peptides and could not be differentiated based on MS/MS analysis alone were grouped to satisfy the principles of parsimony. Label-free spectral counting was used to determine relative differences in the IgG control and IP samples.

### Statistics and reproducibility

Statistical significance was determined using the Student's *t*-test when comparing the mean of 2 groups. Differences shown in the figures were presented as the mean ± standard error of the mean. All tests were 2-sided, and *P* value <0.05 was considered to be statistically significant. All the statistics were performed using GraphPad Prism 8.0. All the image quantifications were done by the Image-Pro (version 7.0). All experiments were repeated thrice and yielded consistent results; the representative results were shown. Unless specified otherwise, all imaging experiments were repeated independently for at least 3 times, and quantification was done based on 5 high magnification field views.

## Results

### New isoform SIGIRR^ΔE8^ is shorter and possesses new sequence at C-terminus

**FIG. 1.**
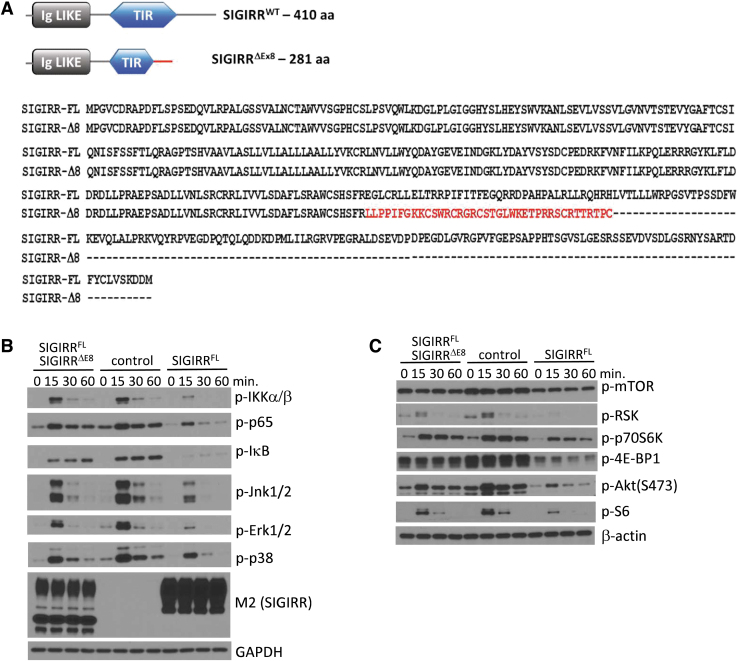
New isoform SIGIRR^ΔE8^ is shorter and possesses new sequence at C-terminus. **(A)** Schematic representation of SIGIRR^FL^ and SIGIRR^ΔE8^ amino acid sequences. In *red* is the new sequence that is formed as a result of the frame shift, unique to SIGIRR^ΔE8^. **(B–C)** Western blot analysis of WCL of 293-IL-1R cells expressing SIGIRR^FL^ or SIGIRR^FL^ and SIGIRR^ΔE8^ stimulated with IL-1β for the indicated times. The experiments were repeated thrice and yielded consistent results; the representative results are shown. IL, interleukin; SIGIRR, single immunoglobulin IL-1-related receptor; WCL, whole cell lysates.

Through analysis of RNA sequencing data from 68 cases of normal and colorectal cancer pairs, we have previously found that SIGIRR^ΔE8^ is increased in the cancer tissue in about 15% of the cases (Zhao et al, 2015). Notably, the skipping of exon 8 results in a frameshift to the sequences coded by the succeeding exons, which replaces part of the intracellular domain with a short peptide sequence unique to SIGIRR^ΔE8^ (Fig. 1A). The resulting protein is shorter and contains only 281 aa. Overexpression of SIGIRR^ΔE8^ reduced the ability of full-length SIGIRR to inhibit IL-1β-induced NFκB, MAPK (Fig. 1B), and mammalian target of rapamycin (mTOR) (Fig. 1C) signaling pathways.

### New sequence of SIGIRR^ΔE8^ serves as an ER retention signal

**FIG. 2.**
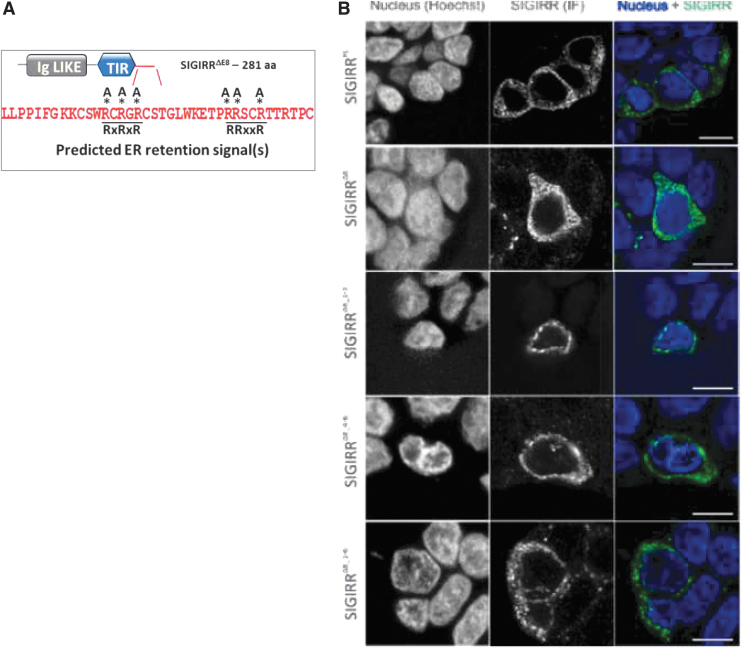
New sequence of SIGIRR^ΔE8^ serves as an ER retention signal. **(A)** Schematic representation of SIGIRR^ΔE8^. The unique peptide (in *red*) contains several arginine (R) residues that conform to the arginine-based ER retention signal motif. **(B)** Immunofluorescence micrographs showing the cellular location of HA-tagged SIGIRR^FL^, SIGIRR^EΔ8^, and SIGIRR^EΔ8^ ER mutants. The expression vectors were transfected into HT-29 cells. The experiments were repeated thrice and yielded consistent results; the representative results are shown. ER, endoplasmic reticulum; HA, hemagglutinin.

Unlike full-length SIGIRR, which localizes predominantly on the basal lateral membrane of colonic epithelial cells, SIGIRR^ΔE8^ is primarily retained in the ER (Zhao et al, 2015). The unique peptide contains several arginine residues that conform to the arginine-based ER retention signal motif (Pan et al, 2015) (Fig. 2A). Therefore, we hypothesized that the arginine residues found in the C-terminus of SIGIRR^ΔE8^ serve as an ER retention signal that prevents SIGIRR^ΔE8^ from trafficking to the membrane. We mutated the predicated sites by site-directed mutagenesis changing the arginines to alanines. Mutated forms of SIGIRR^ΔE8^ (SIGIRR^ΔE8 1–3,3–6,1–6^) showed decreased ER and increased membrane localization compared to unmutated one (Fig. 2B).

**FIG. 3.**
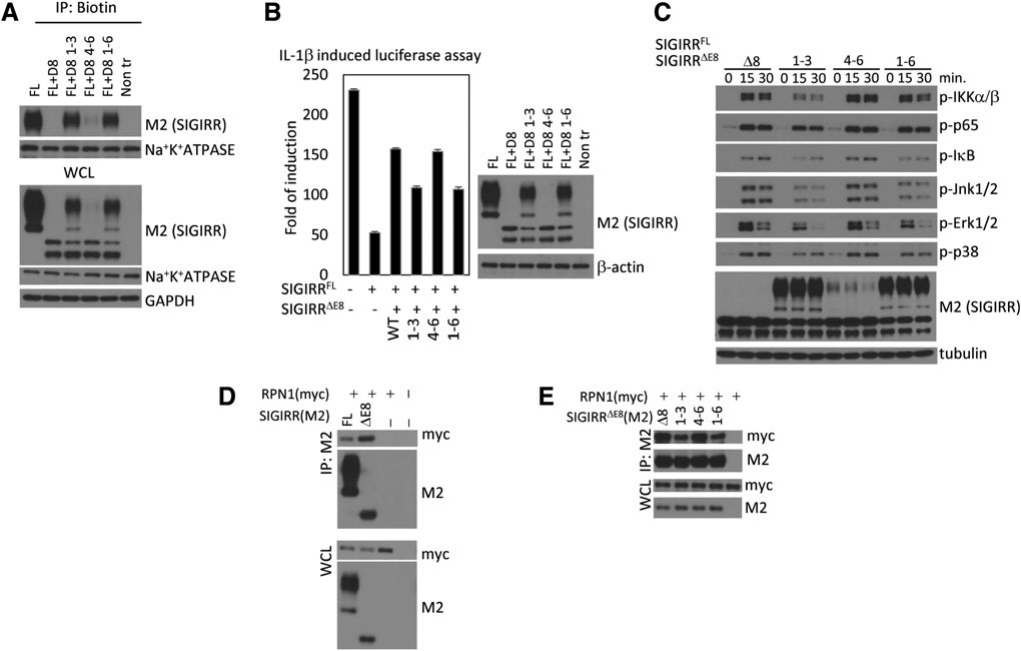
ER retention signal is important for SIGIRR^FL^ inhibition and RPN1 interaction. **(A)** HeLa cells were transfected with equal amounts of SIGIRR^FL^ and SIGIRR^ΔE8^. Transfected cells were subjected to *in situ* biotinylation assay followed by Western blot analysis. **(B)** Equal amounts of SIGIRR^FL^ and SIGIRR^ΔE8^ were transfected into HeLa cells together with NFκB-dependent luciferase construct. Transfected cells were subjected to the luciferase assay (*left panel*) and Western blot (*right panel*). Error bar represents SEM of 3 technical replicates. **(C)** Western blot analysis of WCL of 293-IL-1R cells expressing SIGIRR^FL^ or SIGIRR^FL^ and SIGIRR^ΔE8^ or its ER mutants stimulated with IL-1β for the indicated times. **(D, E)** HeLa cells were cotransfected with myc-tagged RPN1 and M2 flag-tagged SIGIRR^FL^ and SIGIRR^ΔE8^ **(D)** or SIGIRR^FL^ and SIGIRR^ΔE8^ ER mutants. **(E)** Transfected cells were lysed, and the lysates were immunoprecipitated with anti-FLAG antibody followed by Western blot analysis. The experiments were repeated thrice and yielded consistent results; the representative results are shown. NFκB, nuclear factor kappa B; RPN1, resident protein ribophorin 1; SEM, standard error of the mean.

In addition, upon mutation the first 3 arginines (1–3) or all of them (1-6), we found that SIGIRR^ΔE8^ lost its ability to completely inactivate full-length SIGIRR (Fig. 3A–C). Loss of the ER retention signal abolished the dominant negative function of SIGIRR^ΔE8^ as it could no longer trap full-length SIGIRR in the cytoplasm. Using biotinylation assay we showed that mutants 1–3 and 1–6 restore membrane localization of full-length SIGIRR (Fig. 3A). By cotransfecting SIGIRR^ΔE8^ or SIGIRR^ΔE8^ mutants together with full-length SIGIRR followed by luciferase assay, we determined that the inhibition of IL-1R signaling by full-length SIGIRR was restored to some degree by SIGIRR^ΔE8^ mutants 1–3 and 1–6 (Fig. 3B). Similarly, the ability of full-length SIGIRR to inhibit IL-1β-induced NFκB and MAPK activation was restored when cotransfected with mutants 1–3 and 1–6 (Fig. 3C). Together these results indicate that first 3 arginines are required for the ER retention of SIGIRR^ΔE8^.

### ER retention signal is important for RPN1 interaction

Previously we have shown that the interaction with RPN1 also contributed to the ER retention, since SIGIRR^ΔE8^ exhibited increased interaction with RPN1 (Zhao et al, 2015) (Fig. 3D). Therefore we tested if the ER retention signal is important for this interaction. Interestingly we have found that SIGIRR^ΔE8^ mutants 1–3 and 1–6 showed reduced ability to interact with RPN1 when overexpressed (Fig. 3E).

### SIGIRR^ΔE8^ interacts with a mitochondrial protein ATP5A1

**FIG. 4.**
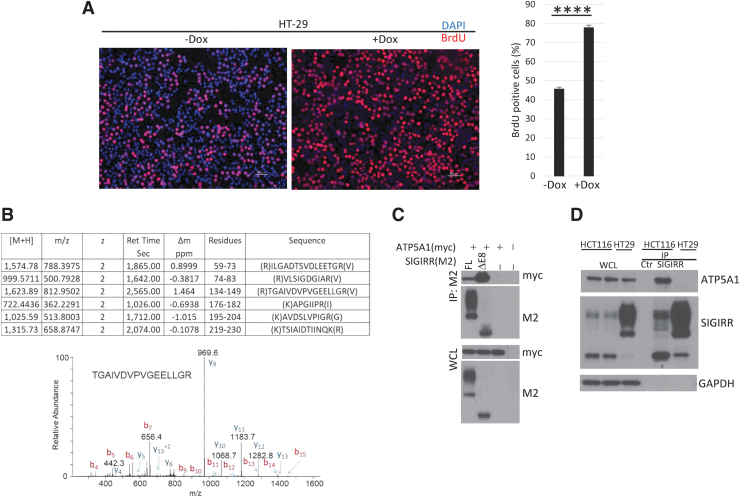
SIGIRR^ΔE8^ interacts with a mitochondrial protein ATP5A1. **(A)** Engineered HT-29 cells inducibly expressing SIGIRR^ΔE8^ were incubated with 10 μM of BrdU for 6 h followed by fixation with PFA and immunofluoresence staining with anti-BrdU antibody. **(B)** HA pull-down experiment was performed from cells expressing a HA-tagged SIGIRR. These proteins were fractionated on an SDS-Page gel, the fractionated proteins were digested with trypsin, and the digests were analyzed by LC-MS/MS analysis. The protein, ATP5A1, was identified in the SIGIRR pull-down experiments by a total of 6 peptides (*top*). One of these peptides corresponds to the (134)TGAIVDVPVGEELLGR(149) peptide, which was identified as a doubly charged peptide with a mass of 812.950 Da. The CID spectra for this peptide are given above and are dominated by singly charged C-terminal y ions and N-terminal b ions (*bottom*). **(C)** HeLa cells were cotransfected with myc-tagged ATP5A1 and M2 flag-tagged SIGIRR^FL^ and SIGIRR^ΔE8^. Transfected cells were lysed, and the lysates were immunoprecipitated with anti-FLAG antibody followed by Western blot analysis. **(D)** SIGIRR protein was immunoprecipitated from indicated cell lines. Coimmunoprecipitated proteins were resolved on SDS-PAGE followed by Western blot for ATP5A1 and SIGIRR. The experiments (except MS) were repeated thrice and yielded consistent results; the representative results are shown. Data are shown as mean ± SEM. ****P < 0.0001 by 2-tailed *t*-test. Scale bar, 50 μm. CID, collision-induced dissociation; LC-MS/MS, liquid chromatography–mass spectrometry/mass spectrometry; PFA, paraformaldehyde; SDS-PAGE, sodium dodecyl sulfate–polyacrylamide gel electrophoresis.

Next, we wondered whether SIGIRR^ΔE8^ confers other novel function(s) in addition to trapping full-length SIGIRR resulting in loss of SIGIRR's inhibitory effect on TLR/IL-1R signaling. Indeed, the BrdU incorporation showed that SIGIRR^ΔE8^ overexpression resulted in an increased cell proliferation rate of colorectal cell line—HT-29 cells (Fig. 4A). To explore the potential new role of SIGIRR^ΔE8^, we performed mass spectrometry to search for SIGIRR-interacting proteins in HCT116, where predominantly SIGIRR^ΔE8^ is expressed. In this proteome profiling experiment, we identified a mitochondrial protein, the alpha subunit of adenosine triphosphate (ATP) synthase (ATP5A1), as an interacting protein of SIGIRR in colorectal cancer cells (Fig. 4B). We confirmed these results in the overexpression system (Fig. 4C) and also by pulling down endogenous SIGIRR in HCT116 and HT-29 cells. We observed increased interaction in HCT116, where predominantly SIGIRR^ΔE8^ is expressed, compared to HT-29 cells that predominantly express the full-length SIGIRR (Fig. 4D).

ATP5A1 is a subunit of mitochondrial ATP synthase (Godbout et al, 1997), which catalyzes ATP synthesis using an electrochemical gradient of protons across the inner membrane during oxidative phosphorylation. As mitochondrial ATP production is the main energy source for intracellular metabolic pathways, the interaction of SIGIRR^ΔE8^ with ATP5A1 may have a critical impact on cell metabolism, thereby promoting cell growth and tumorigenesis. Notably, we found that SIGIRR^ΔE8^ overexpression indeed increased the expression of a gene set that the gene ontology enrichment analysis classifies as metabolic pathways (Zhao et al, 2015). Interestingly, ATP5A1 has been shown to be a critical modifier of the *Apc*^min^ mediated intestinal tumorigenesis, and ATP5A1 variants have also been reported to associate with colorectal cancer prognosis in humans (Baran et al, 2007). Taken together, these data suggest that SIGIRR^ΔE8^ may exert a direct impact on cell metabolism in addition to its dominant negative role on full-length SIGIRR function.

### SIGIRR^ΔE8^ expression increases cell metabolism

**FIG. 5.**
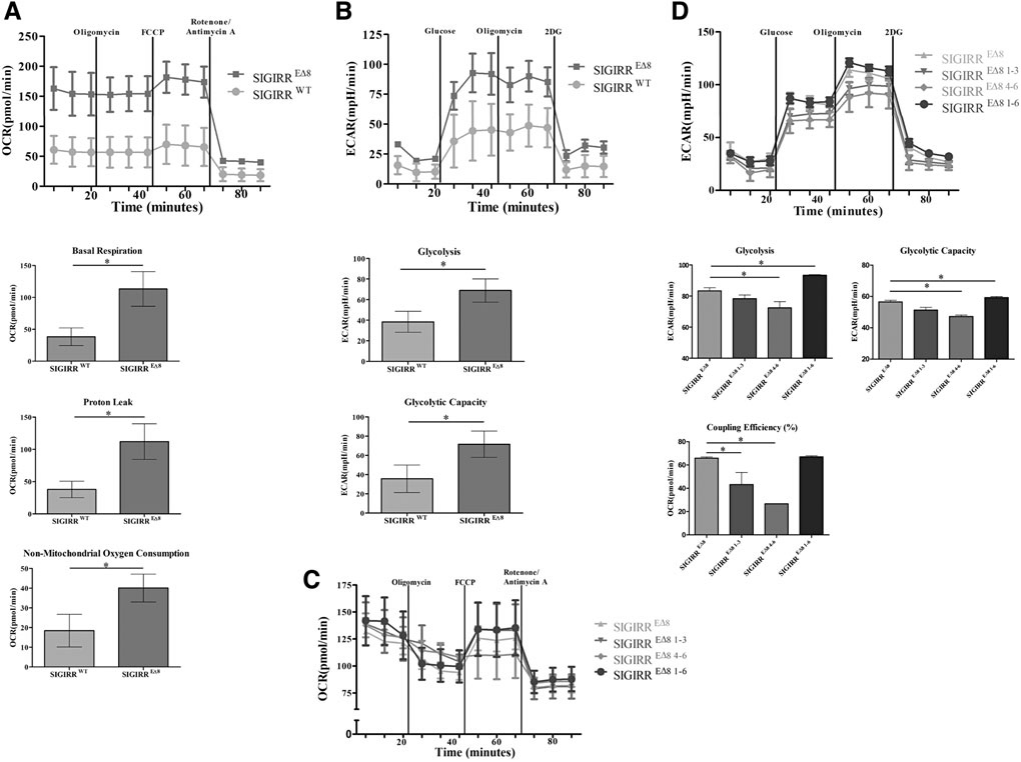
SIGIRR^ΔE8^ expression increases cell metabolism. (**A, B)** Real-time measurement of OCR **(A)** and ECAR **(B)** of the HeLa cells expressing V5-tagged SIGIRR^ΔE8^. **(C, D)** Real-time measurement of OCR **(C)** and ECAR **(D)** of the HeLa cells expressing SIGIRR^ΔE8^ and ER retention mutants of SIGIRR^ΔE8^. Comparison of basal respiration, nonmitochondrial oxygen consumption, proton leak, glycolysis, and glycolytic capacity. Data are shown as mean ± SEM. *P < 0.05 by 2-tailed *t*-test. The experiments were repeated thrice and yielded consistent results; the representative results are shown. ECAR, extracellular acidification rate; OCR, oxygen consumption rate.

As the next step we compared metabolic phenotypes of the cells expressing SIGIRR^ΔE8^ and control ones using the Seahorse XFp Analyzer. To examine the effect of SIGIRR^ΔE8^ on OXPHOS, we performed the mitochondrial respiration test and measured OCR. We found that SIGIRR^ΔE8^ expression significantly increases the respiratory parameters (Fig. 5A), including basal respiration (initial OCR measured before addition of any inhibitors minus nonmitochondrial respiration), proton leak (difference between the ATP-linked OCR and the nonmitochondrial respiration), and nonmitochondrial respiration (OCR after addition of rotenone and antimycin A).

Next we measured the glycolytic parameters, including the glucose metabolism ECAR after addition of glucose; the maximal glycolytic capacity following OXPHOS ATP synthesis with oligomycin; and the nonglycolytic extracellular acidification measured after treatment with 2-DG, an inhibitor of hexokinase II, which catalyzes the first step of glycolysis. We found that SIGIRR^ΔE8^ expression led to significantly higher glycolytic capacity and glycolysis (Fig. 5B).

OCR, as well as ECAR, was increased compared to the control cells (Fig. 5A, B), which suggests that the increased glycolytic flux in SIGIRR^ΔE8^ overexpressing cells did not represent a shift from oxidative to glycolytic metabolism but rather a general increase in metabolic activity. Interestingly, the ER retention mutants of SIGIRR^ΔE8^ (1–3 and 4–6) have reduced OCR and ECAR parameters compared to unmutated SIGIRR^ΔE8^ (Fig. 5C, D). These results indicate that SIGIRR^ΔE8^ impacts on the cell metabolism.

### SIGIRR^ΔE8^ expression promotes tumor growth in xenograft model

**FIG. 6.**
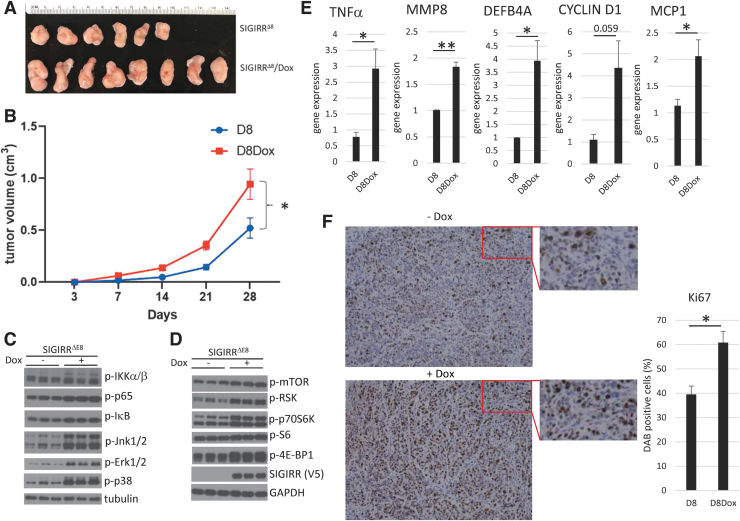
SIGIRR^ΔE8^ expression promotes tumor growth in xenograft model. **(A)** Macroscopic tumor image from mice injected with or without Dox induced SIGIRR^ΔE8^ inducible HT-29 cells (*n* = 6–8 mice). **(B)** Tumor growth kinetics for panel **(A)**. Data are presented as mean ± SEM, *P < 0.05 by 2-way ANOVA followed by Tukey's test. **(C)** Gene expression of tumor tissue. Data are shown as mean ± SEM. *P < 0.05, **P < 0.01 by 2-tailed *t*-test. **(D, E)** Western blot analysis of WCL of tumor tissue. The experiments were repeated thrice and yielded consistent results; the representative results are shown. **(F)** Immunohistochemistry analysis of Ki67 in tumor tissue from mice with indicated treatment. Data are shown as mean ± SEM. *P < 0.05 by 2-tailed *t*-test. Scale bar, 50 μm. ANOVA, analysis of variance.

We have identified a classical colon cancer cell line (HT-29) that predominantly expresses the full-length SIGIRR. We introduced SIGIRR^ΔE8^ in an inducible manner, and we tested the impact of SIGIRR^ΔE8^ on tumor growth. The engineered cells were injected into the flanks of the immune deficient NSG mice to establish a xenograft model. The xenograft was monitored to compare the kinetics of tumor growth for 28 days. We found that SIGIRR^ΔE8^ significantly accelerated the growth curve (Fig. 6A, B). The activation of both NFκB/MAPK and mTOR pathways and associated gene expression were increased in the tumors overexpressing SIGIRR^ΔE8^ compared to the controls (Fig. 6C–E). Consistently, increased mTOR activity in the xenograft model led to higher cell proliferation as Ki67 positive cells were increased in the tissue with SIGIRR^ΔE8^ overexpression (Fig. 6F). Taken together, these results show that SIGIRR^ΔE8^ promotes the tumor growth.

## Discussion

We have shown previously that SIGIRR^ΔE8^ expression is frequently increased in human colorectal cancer (Zhao et al, 2015). *In vitro* analysis indicates that SIGIRR^ΔE8^ traps full-length SIGIRR in the cytoplasm and that membrane localization is required for full-length SIGIRR to function. Considering that cytoplasmic expression of SIGIRR is correlated with poor tumor grade, we hypothesize that expression of SIGIRR^ΔE8^ promotes the growth of human colorectal cancer. We tested this hypothesis through xenograft mouse model.

SIGIRR^ΔE8^ exhibits reduced membrane localization in overexpression system compared to full-length SIGIRR. It also shows increased interaction with ER RPN1, suggesting increased ER retention. Moreover, SIGIRR^ΔE8^ interacts with full-length SIGIRR and prevents it from trafficking to the plasma membrane. Importantly, the membrane localization is required for proper function of the full-length SIGIRR. Consistently, SIGIRR localized predominantly to the cytoplasm and colocalized with RPN1 in the colorectal cancer tissues while it is primarily distributed along the basal lateral membrane in normal colonic epithelial cells.

It was therefore important to elucidate the mechanism underlying the ER retention of SIGIRR. A major difference between full length and SIGIRR^ΔE8^ is the unique C-terminus generated by the frameshift resulting from exon 8 skipping. The unique peptide contains several arginine residues that conform to the arginine-based ER retention signal motif. In current study size we showed that the arginine residues found in the C-terminus of SIGIRR^ΔE8^ serves as an ER retention signal that prevents SIGIRR^ΔE8^ from trafficking to the membrane.

In the later part of our article, we determined the impact of SIGIRR^ΔE8^ expression on cell metabolism. It is widely accepted that cancer cells exhibit abnormal metabolism characterized by increased aerobic glycolysis (Warburg Effect), which has been shown to be essential for cancer cell survival and proliferation. However, in addition to increase in ECAR, we also observed a rise of OCR, suggesting that the increased glycolytic flux in SIGIRR^ΔE8^ expressing cells did not represent a further shift from oxidative to glycolytic metabolism, but rather a general increase in metabolic activity.

While full-length SIGIRR negatively regulates mTOR activation, SIGIRR^ΔE8^ interacts with the ATP synthase in the colorectal cancer cells. Considering that SIGIRR predominantly localizes to the ER in the cancer cells, it is possible that the SIGIRR-ATP synthase interaction occurs at the mitochondrial-ER interface. Mitochondria-ER interaction is implicated in mitochondria fragmentation, which compromises oxidative phosphorylation and promotes the use of aerobic glycolysis pathways. In support of this, SIGIRR^ΔE8^ overexpression impinged the metabolic pathways through gene induction. We showed that SIGIRR^ΔE8^ expression promotes the metabolic shift through upregulation of mTOR (suppressing full-length SIGIRR) and/or dysregulation of mitochondrial function (interacting with ATP5A1) to promote survival and proliferation of colon cancer cells.

In summary, we have shown that SIGIRR^ΔE8^ promotes human CRC through 2 synergistic mechanisms: (1) it relieves the inhibition on TLR/IL-1R signaling through its dominant negative effect on full-length SIGIRR, enhancing the activation of NFκB and mTOR. The unique sequence of SIGIRR^ΔE8^ serves as ER retention signal; (2) it interacts with ATP synthase and increases the cell metabolism. While NFκB activation generates an inflammatory microenvironment that favors tumor growth, mTOR activation increases the translation of cell survival genes (cyclin D1, Bcl-xL, and COX2).

Understanding how SIGIRR exerts its inhibitory role on the signaling events mediated by TLR/IL-1R has important clinical implications—it may lead to significant advances in the field of inflammation and colonic tumorigenesis and help us to determine the potential of SIGIRR as a target for developing anti-inflammatory and anticancer drugs.
